# Detection of stretch reflex onset based on empirical mode decomposition and modified sample entropy

**DOI:** 10.1186/s42490-019-0023-y

**Published:** 2019-09-26

**Authors:** Mingjia Du, Baohua Hu, Feiyun Xiao, Ming Wu, Zongjun Zhu, Yong Wang

**Affiliations:** 1 School of Mechanical Engineering, Hefei University of Technology, No. 193 Tunxi Road, Hefei, 230009 China; 2 Department of Rehabilitation Medicine, Anhui Provincial Hospital, No. 1 Swan Lake Road, Hefei, 230001 China; 3 Acupuncture and Rehabilitation Department, The First Affiliated Hospital of Anhui University of Chinese Medicine, No. 117 Meishan Road, Hefei, 230031 China

**Keywords:** Spasticity, Stretch reflex onset, Surface electromyography, Empirical mode decomposition, Modified sample entropy

## Abstract

**Background:**

Accurate spasticity assessment provides an objective evaluation index for the rehabilitation treatment of patients with spasticity, and the key is detecting stretch reflex onset. The surface electromyogram of patients with spasticity is prone to false peaks, and its data length is unstable. These conditions decrease signal differences before and after stretch reflex onset. Therefore, a method for detecting stretch reflex onset based on empirical mode decomposition denoising and modified sample entropy recognition is proposed in this study.

**Results:**

The empirical mode decomposition algorithm is better than the wavelet threshold algorithm in denoising surface electromyogram signal. Without adding Gaussian white noise to the electromyogram signal, the stretch reflex onset recognition rate of the electromyogram signal before and after empirical mode decomposition denoising was increased by 56%. In particular, the recognition rate of stretch reflex onset under the optimal parameter of the modified sample entropy can reach up to 100% and the average recognition rate is 93%.

**Conclusions:**

The empirical mode decomposition algorithm can eliminate the baseline activity of the surface electromyogram signal before stretch reflex onset and effectively remove noise from the signal. The identification of stretch reflex onset using combined empirical mode decomposition and modified sample entropy is better than that via modified sample entropy alone, and stretch reflex onset can be accurately determined.

## Background

Muscle spasticity is an intermittent or persistent involuntary excessive movement of the skeletal muscle caused by an upper motor neuron injury [1]. Spasticity is clinically manifested by an increase in passive stretching resistance, i.e., an increase in muscle tension, and resistance increases with passive stretching speed [2]. Accurate spasticity assessment provides an objective evaluation index for the rehabilitation treatment of patients with spasticity [3]. The modified Ashworth scale is the most widely used method for assessing spasticity in the clinical setting. It is simple and easy to implement without the aid of instruments. However, the modified Ashworth scale exhibits strong subjectivity; thus, it cannot meet the requirements of accurate spasticity assessment [4].

The tonic stretch reflex threshold is currently recognized as the most effective and consistent value for assessing spasticity [5]. The stretch reflex threshold represents the joint angle at which antagonist muscles or motor neurons begin to contract when the affected limbs of the subject are stretched passively [6]. The starting point that corresponds to the beginning of contraction of antagonistic muscles or motor neurons is called stretch reflex onset [7]. Surface electromyogram (EMG) is a nonlinear and nonstationary signal obtained from electrodes attached on the skin surface of muscles [8]. The spasticity of a patient can be analyzed, and the nerve components during an increase in muscle tension can be distinguished by analyzing surface EMG signals. Stretch reflex onset detection using surface EMG signal is a prerequisite and basic step in biomedical research and clinical diagnosis, such as gait recognition, and automatic prosthetic control [9–11]. It is crucial for detecting the stretch reflex threshold of muscles when assessing spasticity [12–14].

At present, the commonly used stretch reflex onset detection method is based on experience or the standard deviation (SD) of surface EMG signal [1, 15]. Experience is too subjective [12], and the SD of surface EMG signal is susceptible to the baseline activity of such signal [16]. SD cannot achieve exact onset detection give the low signal-to-noise ratio (SNR) of surface EMG signal in patients with spasticity [17]. Detection methods based on time-domain analysis and statistical characteristics, such as the Teager–Kaiser energy operator and the maximum likelihood method, have been effectively applied in recent years. The Teager–Kaiser energy operator considers the amplitude and instantaneous frequency of signals to improve the recognition rate of onset detection; however, it requires an extremely high SNR [18]. The maximum likelihood method is commonly used for the onset detection of signals with low SNR (SNR = 1); however, it is only suitable for processing Gaussian noise [19]. Yang et al. combined Teager–Kaiser energy operator with an image enhancement technique and adopted morphological close operators to realize the accurate onset detection of pathological surface EMG signals with weak noise [20].

Sample Entropy is a method based on approximate entropy that is used to measure the complexity of time series. Sample entropy has been applied in evaluating the complexity of physiological time series and in diagnosing pathological state [21]. Zhou et al. used sample entropy in the onset detection of muscle activities and achieved good recognition effect in an environment with background burr noise [22]. However, Sample entropy is highly dependent on data length, and stability is hardly guaranteed when data length is short [23–26]. Compared with the standard sample entropy, the modified sample entropy exhibits stronger dependence on data length and has a smaller fluctuation in entropy value, making the former more suitable for processing short-term surface EMG signals [27]. Considering the poor quality of surface EMG signals in patients with spasticity, preprocessing should be performed before the stretch reflex onset detection of the modified sample entropy [28].

Empirical mode decomposition is the adaptive decomposition of a signal into several intrinsic mode functions in accordance with the time-scale characteristics of data without presetting any basis function [29]. The empirical mode decomposition method proposed by Huang et al. can be applied to any type of signal decomposition and exhibits evident advantages in dealing with nonlinear and nonstationary EMG signals [30, 31]. Zhou et al. combined empirical mode decomposition with soft thresholds to eliminate three types of common noise in surface EMG signal: power line interference, Gaussian white noise, and baseline drift. The empirical mode decomposition denoising has been proven to be better than those of other digital filters, such as traditional infinite impulse response filters [32].

Given the pathological symptoms of patients with spasticity, abnormal overexcitement leads to discharges of motor units, and consequently poor quality of surface EMG signals. The surface EMG of patients with spasticity is prone to unconscious and involuntary false peaks, which cause a slight difference in surface EMG signal before and after the stretch reflex onset. Moreover, a false EMG peak is mixed into the mutant signal after the stretch reflex onset [33]. Therefore, a stretch reflex onset detection method based on empirical mode decomposition denoising and modified sample entropy recognition is proposed in the current study. First, the denoising effects of the empirical mode decomposition and wavelet threshold algorithms are compared through various evaluation indices. Then, the surface EMG signal is decomposed via empirical mode decomposition, and the effective intrinsic mode function is extracted in accordance with the correlation coefficient between each order of the intrinsic mode function and the original signal. The soft threshold is set to denoise the signal on the basis of the surface EMG signal of the subjects in resting state. Lastly, stretch reflex onset is identified using the modified sample entropy. Gaussian white noise with SNRs of 0, 5, 10, 15 and 20 dB is added. The accuracy and robustness of this method and those of the modified sample entropy are compared.

## Results

### Analysis of empirical mode decomposition denoising effect

The semi-synthetic surface EMG signals of 10 patients with spasticity were selected and compared with those of empirical mode decomposition denoising via the wavelet threshold denoising method. Among these signals, the wavelet basis selected the sym4 basis function, which is relatively similar to the surface EMG signals. Three evaluation indices were cited to compare the denoising effects of the two methods, namely, SNR, root mean square error (*R*), and the correlation coefficient (*P*).

Where SNR, *R*, and *P* are expressed as follows:

1 $$ SNR=101g\left(\frac{\sum \limits_{i=1}^N{x}_i(t)}{\sum \limits_{i=1}^N{\left({x}_i(t)-{y}_i(t)\right)}^2}\right), $$

2 $$ R=\sqrt{\frac{1}{N}\sum \limits_{i=1}^N{\left({x}_i(t)-{y}_i(t)\right)}^2}, $$

3 $$ P=\frac{\operatorname{cov}\left({x}_i(t),{y}_i(t)\right)}{\sqrt{D\left({x}_i(t)\right).\kern0.5em D\left({y}_i(t)\right)}} $$

*x*_*i*_*(t)* is the original signal, *y*_*i*_*(t)* is the signal after de-noising, and *N* is the number of sampling points. *Cov*() is the covariance function, *D*() is the variance function.

When SNR is higher, the signal denoising effect is better; when the value of *R* is smaller, the coincidence degree between the denoised and original signals is higher; when the value of *P* is larger, the correlation between the denoised and original signals is stronger. The semi-synthetic surface EMG signals of the 10 subjects were selected, and the evaluation indices were obtained using the two denoising methods (Table 1).

**Table 1:** The evaluation indexes of empirical mode decomposition and wavelet threshold denoising

Subjects	empirical mode decomposition denoising			Wavelet threshold denoising		
	SNR	R	P	SNR	R	P
S1	26.2047	0.1662	0.9989	11.2057	0.9343	0.9614
S2	22.9088	0.1571	0.9975	8.3944	0.8345	0.9248
S3	18.7250	0.4779	0.9933	9.382	1.4012	0.9407
S4	22.8871	0.8001	0.9975	9.5812	3.702	0.9437
S5	23.3546	0.1599	0.9979	10.153	0.7311	0.9509
S6	22.3851	0.1446	0.9976	7.6112	0.792	0.9092
S7	23.1228	0.2509	0.9976	10.9289	1.0212	0.9588
S8	22.9941	0.1091	0.9976	7.0581	0.6835	0.8962
S9	22.1832	0.2532	0.9971	10.6279	0.9577	0.9558
S10	21.7441	0.2805	0.9967	11.9962	0.8615	0.9679

As shown in Table 1, the SNR and *P* of the empirical mode decomposition denoised signals are higher, *R* is smaller, the *P* value of wavelet threshold denoising fluctuates considerably, and denoising performance is unstable. The experimental results indicate that the empirical mode decomposition algorithm is better than the wavelet threshold algorithm in denoising surface EMG signal. The former exhibits stronger denoising ability and better effect, and it can be used as an effective surface EMG signal denoising method.

### Stretch reflex onset detection after denoising of EMG signals

The semi-synthetic surface EMG signals of 25 patients with spasticity were selected, and the data of each patient were added with 0, 5, 10, 15, and 20 dB Gaussian white noise. The stretch reflex onset detection process based on empirical mode decomposition denoising and modified sample entropy recognition is described as follows.

1. Soft threshold denoising of empirical mode decomposition.
2. The denoised surface EMG signal is processed via frame processing with an active sliding window of fixed length. The frame is shifted to one point to calculate the modified sample entropy of each frame.
3. The entropy value is calculated. If the corresponding entropy value at a certain time is greater than the set soft threshold and 50 consecutive points are greater than the soft threshold, then the moment is judged as the stretch reflex onset.

The sliding window length is 32 ms under the optimal parameters of the modified sample entropy, and the threshold sensitivity value is 0.3–0.45. The sliding window length is 64 ms under the optimal parameters of the modified sample entropy, and the threshold sensitivity value is 0.5–0.6. The sliding window length is 96 ms under the optimal parameters of the modified sample entropy, and the threshold sensitivity value is 0.5–0.65. The stretch reflex onset recognition rate of the modified sample entropy in a noisy environment is provided in Table 2. The stretch reflex onset recognition rate of the modified sample entropy after empirical mode decomposition denoising of the surface EMG signals is presented in Table 3. After empirical mode decomposition denoising of the surface EMG signals, the stretch reflex onset recognition rate was obtained under the optimal parameter of the modified sample entropy is presented in Table 4.

**Table 2:** Stretch reflex onset recognition rate of modified sample entropy under noise environment

	SNR = 0	SNR = 5	SNR = 10	SNR = 15	SNR = 20
32 ms	0.32	0.08	0.16	0.20	0.20
64 ms	0.48	0.20	0.44	0.56	0.52
96 ms	0.48	0.24	0.44	0.52	0.44

**Table 3:** Stretch reflex onset recognition rate of modified sample entropy after denoising

	SNR = 0	SNR = 5	SNR = 10	SNR = 15	SNR = 20
32 ms	0.84	0.36	0.36	0.52	0.44
64 ms	0.88	0.64	0.76	0.76	0.76
96 ms	0.80	0.64	0.76	0.68	0.72

**Table 4:** Stretch reflex onset recognition rate under Optimal Parameters after denoising

	SNR = 0	SNR = 5	SNR = 10	SNR = 15	SNR = 20
32 ms	0.96	0.84	0.92	0.88	0.88
64 ms	1	0.92	1	0.92	0.88
96 ms	1	0.88	1	0.96	0.84

**Fig. 1:**
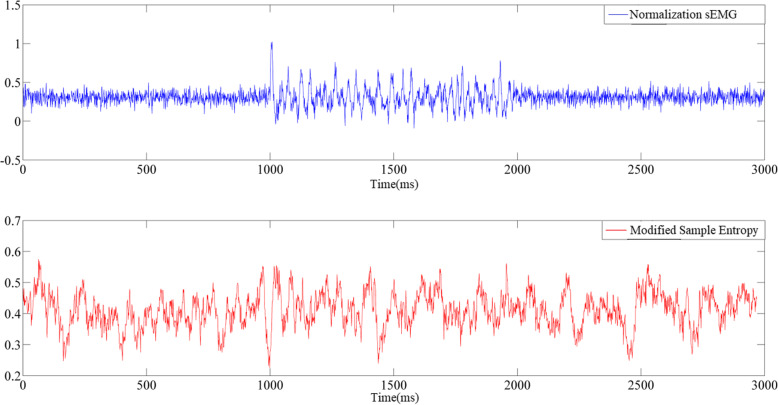
The modified sample entropy under noise (SNR = 20 dB) environment

**Fig. 2:**
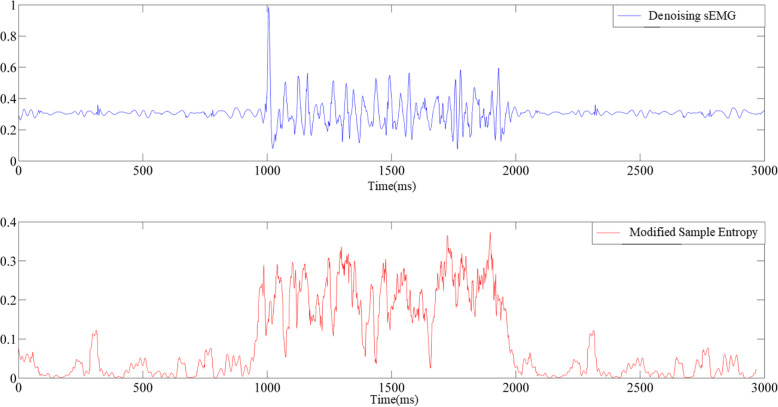
The modified sample entropy after denoising

As shown in Figs. 1 and 2, the semi-synthetic surface EMG signals of a patient with the modified Ashworth scale level of 1+ were selected, and Gaussian white noise that corresponded to an SNR of 20 dB was added. The modified sample entropy values before and after denoising were compared. The difference in the EMG signals before and after the stretch reflex onset increased after empirical mode decomposition denoising as shown by the signal time-domain and modified sample entropy diagrams. As indicated in Tables 2, 3, and 4, the stretch reflex onset recognition rate gradually increased. The average stretch reflex onset recognition rate of the modified sample entropy in a noisy environment is 35%, and the average stretch reflex onset recognition rate of the modified sample entropy after empirical mode decomposition denoising of the surface EMG signals is 66%. The average stretch reflex onset recognition rate under the optimal parameters of the modified sample entropy is 93% after empirical mode decomposition denoising of the surface EMG signals. Without adding Gaussian white noise to the EMG signals, the stretch reflex onset recognition rate of the EMG signals before and after empirical mode decomposition denoising was increased by 56%. The experimental results showed that the stretch reflex onset recognition rate was significantly increased after empirical mode decomposition denoising. In particular, the stretch reflex onset recognition rate can reach up to 100% and the average recognition rate is 93% under the optimal parameters of the modified sample entropy. However, one study [34] reported that the stretch reflex onset recognition rate of the Teager–Kaiser energy operator is 88%. For the signals with added Gaussian noise, the stretch reflex onset recognition rate was significantly increased after empirical mode decomposition denoising. This result indicates that the empirical mode decomposition algorithm can effectively remove noise interference from surface EMG signals and exhibits good anti-noise performance.

## Discussion

Objective evaluation indices are provided for the rehabilitation treatment of patients with spasticity through the stretch reflex onset recognition method of combined empirical mode decomposition denoising and modified sample entropy recognition. The empirical mode decomposition algorithm can remove noise from surface EMG signals and retain useful information, and the soft threshold of the empirical mode decomposition algorithm expands the difference between the signals before and after the stretch reflex onset. Although the modified sample entropy can recognize stretch reflex onset, the poor quality of surface EMG signals in patients with spasticity leads to a slight difference in surface EMG signals before and after the stretch reflex onset. Moreover, false EMG peaks are mixed into the mutation signal after the stretch reflex onset. Thus, EMG signals must be preprocessed before the stretch reflex onset detection. For the surface EMG signals of patients with spasticity, the performance of the stretch reflex onset detection method using combined empirical mode decomposition and the modified sample entropy algorithm is better than that of the modified sample entropy algorithm alone. The former is more suitable for clinical applications.

### Analysis of results

We evaluated the denoising effect of the empirical mode decomposition and wavelet threshold algorithms through three evaluation indicators. The SNR and *P* values after empirical mode decomposition denoising were higher than the results after wavelet threshold denoising. The root mean square error value was lower than the results after wavelet threshold denoising. The experimental results show that the empirical mode decomposition algorithm is better than the wavelet threshold algorithm in denoising surface EMG signals. The former exhibits stronger denoising ability and better effect, and it can be used as an effective surface EMG signal denoising method. By using the stretch reflex onset detection method of combined empirical mode decomposition denoising and modified sample entropy recognition, the stretch reflex onset recognition rate can reach up to 100% and the average recognition rate is 93% under the optimal parameters of the modified sample entropy. The stretch reflex onset recognition rate of combined empirical mode decomposition denoising and modified sample entropy recognition is considerably higher than that of modified sample entropy recognition alone. The combination of empirical mode decomposition and the modified sample entropy can be applied to detect stretch reflex onset in patients with spasticity.

### Study limitations

This study only analyzed the stretch reflex onset of surface EMG signals. The quantitative assessment of upper limb flexor spasticity is determined on the basis of the tension stretch reflex threshold, which is determined on the basis of surface EMG and joint angle signals [13, 14]. The tensile stretch reflex threshold depends on speed. In this experiment, a certain velocity fluctuation occurs when the doctor uniformly stretches a patient’s limbs. We plan to design an isokinetic instrument that can measure spasticity and eliminate the influence of velocity fluctuation. Surface EMG signals are used in this study to analyze the stretch reflection threshold. We can also add a linear acceleration sensor in the experiment to determine the stretch reflection threshold through multiple parameters. The sample size used in this experiment is small. In the future, the sample size of patients with different spasticity grades will be increased, and experiments will be conducted at different stretch speeds to further comprehensively verify the reliability of the stretch reflex onset detection based on combined empirical mode decomposition and the modified sample entropy.

### Clinical implications

The upper limb spasticity assessment system based on surface EMG signals is simple and convenient to use in spasticity assessment in the clinical setting. The empirical mode decomposition and modified sample entropy algorithm exhibit satisfactory clinical applicability for the pathological surface EMG signals of patients with spasticity. Accurate spasticity assessment provides an objective evaluation index for the rehabilitation treatment of patients with spasticity. On the one hand, it can objectively reflect the rehabilitation status of stroke survivors. On the other hand, it can assist in adjusting treatment programs and formulating personalized rehabilitation programs. The realization of clinically accurate stretch reflex onset and the measurement of stretch reflex intensity can help understand the spasticity and pathological mechanism of increased muscle tension, track the rehabilitation of patients with spasticity, and screen targeted reasonable treatment programs.

## Conclusion

A stretch reflex onset detection method based on combined empirical mode decomposition and modified sample entropy was proposed to address the problem of EMG signals in patients with spasticity. On the basis of empirical mode decomposition denoising and modified sample entropy recognition experiments, the following conclusions were drawn from this study. 1) The empirical mode decomposition algorithm is better than the wavelet threshold algorithm in denoising surface EMG signals because it can retain useful information and effectively remove noise from surface EMG signals. 2) Gaussian white noise with different SNRs was added to the original signal, and the results showed that empirical mode decomposition exhibits good anti-noise performance and its denoising capability is helpful in the stretch reflex onset detection. The stretch reflex onset recognition rate of combined empirical mode decomposition denoising and modified sample entropy recognition is considerably higher than that of the modified sample entropy alone. The stretch reflex onset recognition rate under the optimal parameters of the modified sample entropy can reach up to 100% and the average recognition rate is 93%. In summary, the empirical mode decomposition algorithm can effectively remove noise from surface EMG signals, and the stretch reflex onset detection rate of combined empirical mode decomposition and the modified sample entropy is high. Thus, accurate and reliable stretch reflex onset detection can be realized, and a new method for the objective and accurate evaluation of spasticity is provided.

## Methods

### Experimental

The surface EMG signals of biceps brachii during the passive extension of the upper limbs were collected from patients with spasticity. The subject sat upright while the doctor passively stretched the upper limb of the affected side of the subject, allowing the upper limb to adapt to the traction speed. Sudden traction was prevented from causing tension in the subjects that might affect experimental data. At the end of the preparatory activity, the doctor collected the surface EMG signals of each subject at an appropriate uniform speed on the basis of experience, and the process was repeated four times at 2 min intervals. Reacquisition was performed after 1 day. The elbow joint was fixed with one hand, and the wrist joint was held with the other hand. The passive traction of the subject from the maximum bending angle to the maximum extension angle was completed. Spasticity is velocity-dependent; thus, the fluctuation of the stretching speed must be maintained within a certain range during the experiment.

**Fig. 3:**
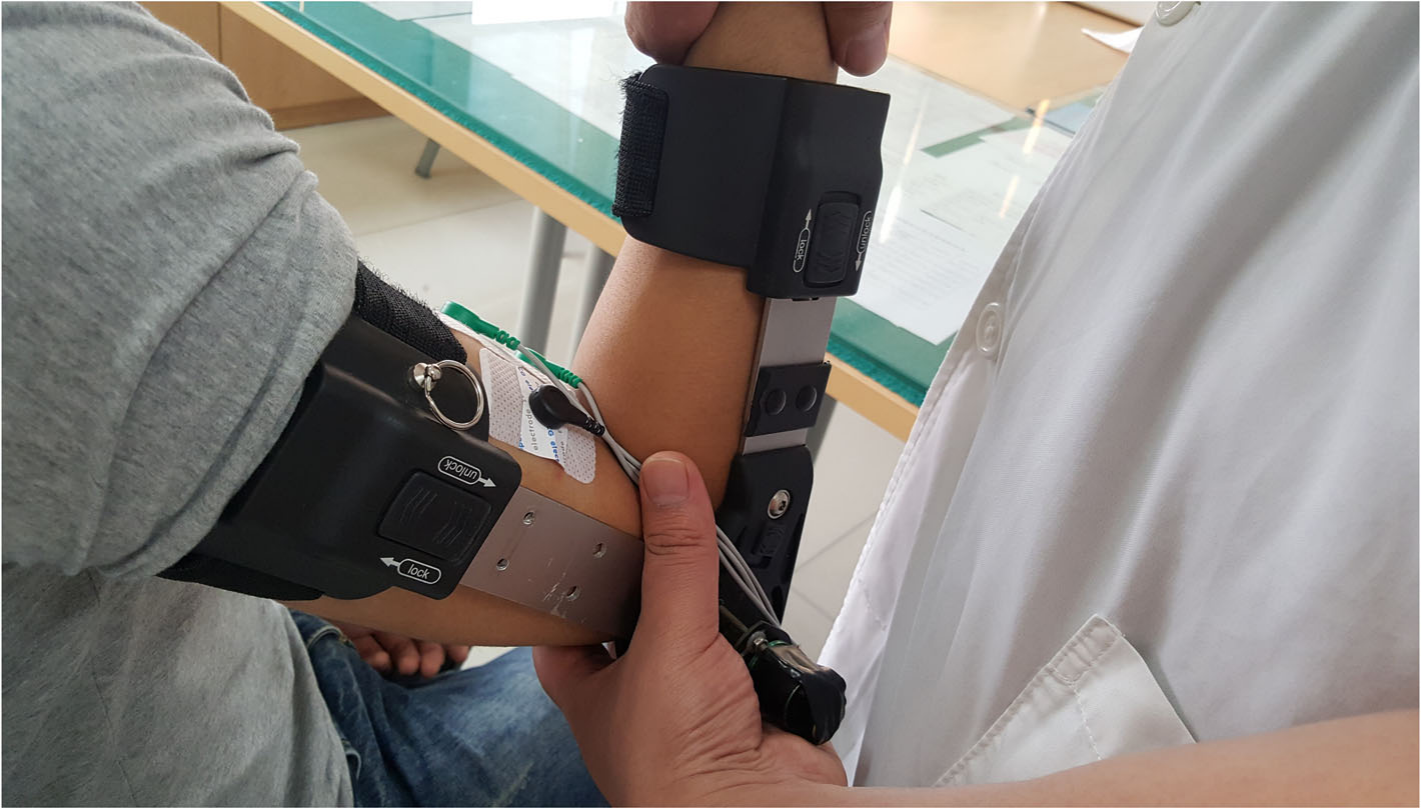
The evaluation process of Spasticity

The EMG signal sensor used in the experiment was based on the hardware circuit and software design of a single-chip microcomputer (STM32). The collected analog signals were A/D converted and then transmitted to the upper computer. The data sampling frequency was 1000 Hz. The positive and negative electrodes of the electrode sheet were attached to the biceps muscle at 2 cm intervals. The reference end was attached to the side of the biceps. The experimental procedure is illustrated in Fig. 3.

A total of 25 eligible stroke survivors from the Anhui Provincial Hospital were selected for the experiment. The study was approved by the Anhui Provincial Hospital Ethics Committee, and all the participants provided informed consent. Among the subjects, 11 belonged to the modified Ashworth scale 1, 7 to the modified Ashworth scale 1+, and 7 to the modified Ashworth scale 2.

Inclusion criteria: patients with upper limb flexor spasticity caused by stroke; can sit independently; an elbow flexion and extension range of at least 90°; clear consciousness, without serious cognitive and audiovisual impairment, can cooperate with the examination to complete simple instructions.

Exclusion criteria: patients with central nervous system disorders, such as multiple sclerosis and other diseases that may cause spasticity of the limbs; patients with diseases that can affect the movement of the elbow joints of the upper limbs, such as fracture of the upper limbs; patients with the modified Ashworth scale grades of 0, 3, and 4.

### Empirical mode decomposition

The empirical mode decomposition method proposed by N.E. Huang is based on the time-scale characteristics of data for signal decomposition without setting any basis function [31]. The original signal is decomposed into a number of narrow-band components, and each component is called intrinsic mode function. The decomposition result consists of several intrinsic mode functions and one residual signal.

4 $$ s(t)=\sum imf\kern0.5em {}_i(t)\kern0.5em +{r}_n(t), $$

Each intrinsic mode function should meet the following conditions [31]:

1. The number of extreme points and zero crossings in the entire dataset should not be more than one.
2. The mean values of the upper and lower envelopes should be zero at any point.

However, actual signals are complex, and thus they do not satisfy the aforementioned conditions. Therefore, Huang made the following assumptions [31]:

1. Any signal is composed of several intrinsic mode functions.
2. Each intrinsic mode function can be linear or nonlinear. The numbers of local zeros and extreme points of each intrinsic mode function are the same, and the upper and lower envelopes are locally symmetric with respect to the time axis.
3. Each signal can contain several intrinsic mode functions at any time. If the modal functions overlap with one another, then a composite signal is formed.

The empirical mode decomposition algorithm assumes that a signal is composed of several intrinsic mode functions. First, all the maximum and minimum points in the original signal *s*(*t*) are extracted. Then the upper envelope *e*_*+*_(*t*) and the lower envelope *e*_*−*_(*t*) are fitted by a cubic spline function. Lastly, the mean values of the upper and lower envelopes are calculated as follows:

5 $$ m(t)=\kern0.5em \frac{e_{+}(t)+e\_(t)}{2}, $$

The difference between the original signal and the mean envelope is calculated as follows:

6 $$ {h}_1^1(t)=s(t)={m}_1(t), $$

If *h*_*1*_(*t*) meets the preceding intrinsic mode function conditions, then *h*_*1*_(*t*) can be used as the first-order intrinsic mode function. If the preceding intrinsic mode function conditions are not met, then *h*_*1*_(*t*) is used as the input and the aforementioned steps are repeated. Assume that *h*_*1*_^*k*^(*t*) meets the preceding intrinsic mode function conditions after *k* times. Then, *h*_*1*_^*k*^(*t*) is used as the first-order intrinsic mode function of the original signal as follows:

7 $$ {c}_1(t)={h}_1^k, $$

The difference between the original signal *s*(*t*) and the first-order intrinsic mode function can be calculated as follows:

8 $$ {r}_1(t)=s(t)-{c}_1(t), $$

*c*_*1*_(*t*) is derived repeatedly to enable *r*_*1*_(*t*) to obtain a second-order component, i.e., *c*_*2*_(*t*). Empirical mode decomposition stops when the *N*-th order intrinsic mode function component *c*_*n*_(*t*) or the remaining amount *r*_*n*_(*t*) is less than a preset value, or when the residual component *r*_*n*_(*t*) is a monotonic function or a constant.

Lastly, empirical mode decomposition decomposes the original signal into *x(t)* as follows:

9 $$ x(t)=\sum \limits_{i=1}^n{c}_i(t)+{r}_i(t) $$

### Surface EMG signal denoising based on empirical mode decomposition

Before the stretch reflex onset surface EMG signal of patients with spasticity exhibits involuntary muscle activities and is mixed with noise during the acquisition process. Consequently, the difference in EMG signal before and after the stretch reflex onset is reduced, and the probability of the stretch reflex onset misjudgment is increased. To eliminate the baseline activity and high-frequency noise components of surface EMG signal, the surface EMG signals of the subjects with non-autonomous activities in resting state were set as the soft threshold. Inspired by the soft threshold function in [12], the soft threshold setting rule is presented as follows:

10 $$ \eta \left({IMF}_{ij}\right)=\mathit{\operatorname{sign}}\left({IMF}_{ij}\right){\left(\left|{IMF}_{ij}\right|-{\lambda}_i\right)}_{+}, $$

where *η* (*IMF*_*ij*_) denotes the *j*-th value in the *i*-th-order intrinsic mode function component after denoising, *IMF*_*i*_ represents the *i*-th-order intrinsic mode function component that should be preprocessed, *λ*_*i*_ indicates that each intrinsic mode function is filtered with two SDs of the surface EMG signal at rest, and (∙)_+_ designates the positive portion.

The SD of surface EMG signals is twice larger than that of baseline signals in the stretch reflex onset [13]. The SD of *IMFi* after the stretch reflex onset is twice greater than that of the intrinsic mode function in resting state. Therefore, pretreatment can effectively eliminate involuntary muscle activities generated during spasticity evaluation, reduce the baseline activity and noise interference of the surface EMG signals before the stretch reflex onset, and expand signal difference before and after the stretch reflex onset.

First, the correlation between each intrinsic mode function component and the original signal *s*(*t*) is calculated, and the effective components are selected in accordance with the correlation results for filtering. The specific processes are as follows:

1. First, the original signal is normalized, and then the signal is decomposed via empirical mode decomposition to obtain *IMF*_*i*_.
2. The correlation between intrinsic mode function and the original signal of each order is calculated. In accordance with the magnitude of the correlation, the effective component is found with the soft threshold of one-tenth of the maximum correlation coefficient, and the pseudo-component is eliminated.
3. Filtering is performed on the effective component, and the signal is reconstructed after filtering to reduce the noise of the original signal.

### Modified sample entropy

Sample entropy reflects the probability of generating new information in a nonlinear dynamic system. The definition of standard sample entropy, shows that any distance greater than the similar tolerance *r* is discarded, and only the number of distances less than or equal to the similarity tolerance *r* is retained. The similarity of vectors is based on the Heaviside function [21, 35–37].

The boundary between various types of signals in an actual physical environment is unclear; thus, determining whether the input sample completely belongs to a certain class is difficult. In addition, the distance fluctuation in an actual surface EMG signal sequence is extremely large, and the slight fluctuations of *d* and *r* may also cause violent fluctuations in the output entropy value. Therefore, the standard sample entropy is insufficient for processing surface EMG signals. From [24], the function is defined on the basis of the sigmoid function as follows:

11 $$ f\left(d,r\right)=\frac{1}{1+\exp \left[\left(d-r\right)/r\right]}, $$

where *d* is less than *r*; and the closer to zero, the closer the output value is to 1. *d* is greater than *r*; and the greater the difference, the closer the output value is to zero. The modified sample entropy is described as follows [21, 24, 38].

For a given *N*-point time series *x*(*n*) = {*x* (1), *x* (2), …, *x*(*N*)}, the modified sample entropy algorithm is calculated as follows.

The continuous *m* values in each frame signal sequence constitute an *m*-dimensional vector, where *X*^*m*^(*i*) = [*x*(*i*); *x*(*i* + 1); …; *x*(*i* + *m* − 1)], 1 ≤ *i* ≤ *N*–*m* + 1.

The distance ${d}_{ij}^m$ between the two vectors *X*^*m*^(*i*) and *X*^m^(*j*) (1 ≤ *j* ≤ *N* − *m*, *j* ≠ *i*) is defined as follows:

12 $$ {d}_{ij}^m=d\left[{X}^m(i),{X}^m(j)\right]=\underset{k\in \left[0,m\kern0.5em 1\right]}{\max}\kern0.5em \left\{\left|x\left(i+k\right)-\left(j+k\right)\right|\right\}, $$

The similarity ${D}_{ij}^m$ between *X*^*m*^(*i*) and *X*^*m*^(*j*) can be calculated by providing a similar tolerance *r* as follows:

13 $$ {D}_{ij}^m=f\left({d}_{ij}^m,r\right)=\frac{1}{1+\exp \left[\left({d}_{ij}^m\right)-r/r\right]} $$

The function ${B}_r^m$ can be calculated as follows:

14 $$ {B}_r^m(i)=\frac{1}{N-m-1}\sum \limits_{j=1,j\ne i}^{N-m}{D}_{ij}^m, $$

15 $$ {B}_r^m=\frac{1}{N-m}\sum \limits_{i=1}^{N-m}{B}_r^m(i), $$

Similarly, the function ${A}_r^m$ can be determined by changing the vector dimension *m* to *m + 1* as follows:

16 $$ {A}_r^m(i)=\frac{1}{N-m-1}\sum \limits_{j=1,j\ne i}^{N-m}{D}_{ij}^{m+1}, $$

17 $$ {A}_r^m=\frac{1}{N-m}\sum \limits_{i=1}^{N-m}{A}_r^m(i), $$

Lastly, the modified sample entropy (mSampEn) can be defined as

18 $$ mSampEn\left(m,r,N\right)=-1n\left[\frac{A_r^m}{B_r^m}\right] $$

### Stretch reflex onset recognition based on the modified sample entropy

The modified sample entropy depends on *N*, *m*, and *r*. In accordance with the empirical value [22, 24], *m* is 2 and *r* is generally (0.1–0.25) SD(*X*). When *X* is the data of each frame, *r* is the local similarity tolerance. When *X* is the total data, *r* is the global similarity tolerance. Studies have confirmed that global similarity tolerance is more suitable for surface EMG signal mutation point detection than local similarity tolerance [22]. A previous study [39] verified that the result was optimal when *r* was 0.25 × SD(*X*). In conclusion, the current study sets *r* as the global similar tolerance, and takes 0.25 × SD(*X*). The sliding window length *N* ranges from 32 ms to 96 ms, with an interval of 32 ms.

The stretch reflex onset of the surface EMG signal of biceps brachii is determined using the modified sample entropy. The surface EMG signal is divided into frames by using a sliding window, and each frame is shifted to one point. The modified sample entropy of each frame signal is calculated as *mSampEn*. The adaptive threshold *Th* is set in accordance with Formula (19). The value of *mSampEn* lower than *Th* is set as zero and the value of *mSampEn* larger than *Th* is retained. The moment is judged as the stretch reflex onset when the value of *mSampEn* is greater than zero at a certain time and 50 consecutive *mSampEn* values are all greater than zero. The threshold sensitivity value *α* ranges from 0.3 to 0.65, with an interval of 0.05.

19 $$ Th=\min (mSampEn)+a\left[\max (mSampEn)-\min (mSampEn)\right], $$

The semi-synthetic surface EMG signal is selected. That is, the stretch reflex onset of the surface EMG signal is known, and thus the stretch reflex onset detection capability of combining empirical mode decomposition denoising with the modified sample entropy is verified. The semi-synthetic surface EMG signals consist of two types. The first type is the surface EMG signal of subjects in resting state, and the second type is the surface EMG signal of subjects with spasticity. The signal duration of each group is 3000 ms. The interval [− 50, + 50] is selected on the basis of the stretch reflex onset that is set in advance. If the detection result is within the interval, then it is considered true detection (TD). If it is beyond the interval, then it is considered false detection (FD). The recognition rate is defined as:

20 $$ recognition\_ rate=\frac{TD}{TD+ FD} $$

## Data Availability

The datasets generated during and/or analyzed during the current study are available from the corresponding author on reasonable request, without breaching participant confidentiality.

## Abbreviations

EMG: Electromyography
SD: Standard deviation
SNR: Signal-to-noise ratios
